# Supplementary material for: SARS-CoV-2 Spike Protein and Its Receptor Binding Domain Promote a Proinflammatory Activation Profile on Human Dendritic Cells
Source: Cells. 2021 Nov 23;10(12):3279. doi: 10.3390/cells10123279 (PMC8699033; doi:10.3390/cells10123279)
Supplement: Supplementary file 1 [file cells-10-03279-s001.zip › cells-1437380 - suppl. material - updated file.pdf]

Supplemental Figures

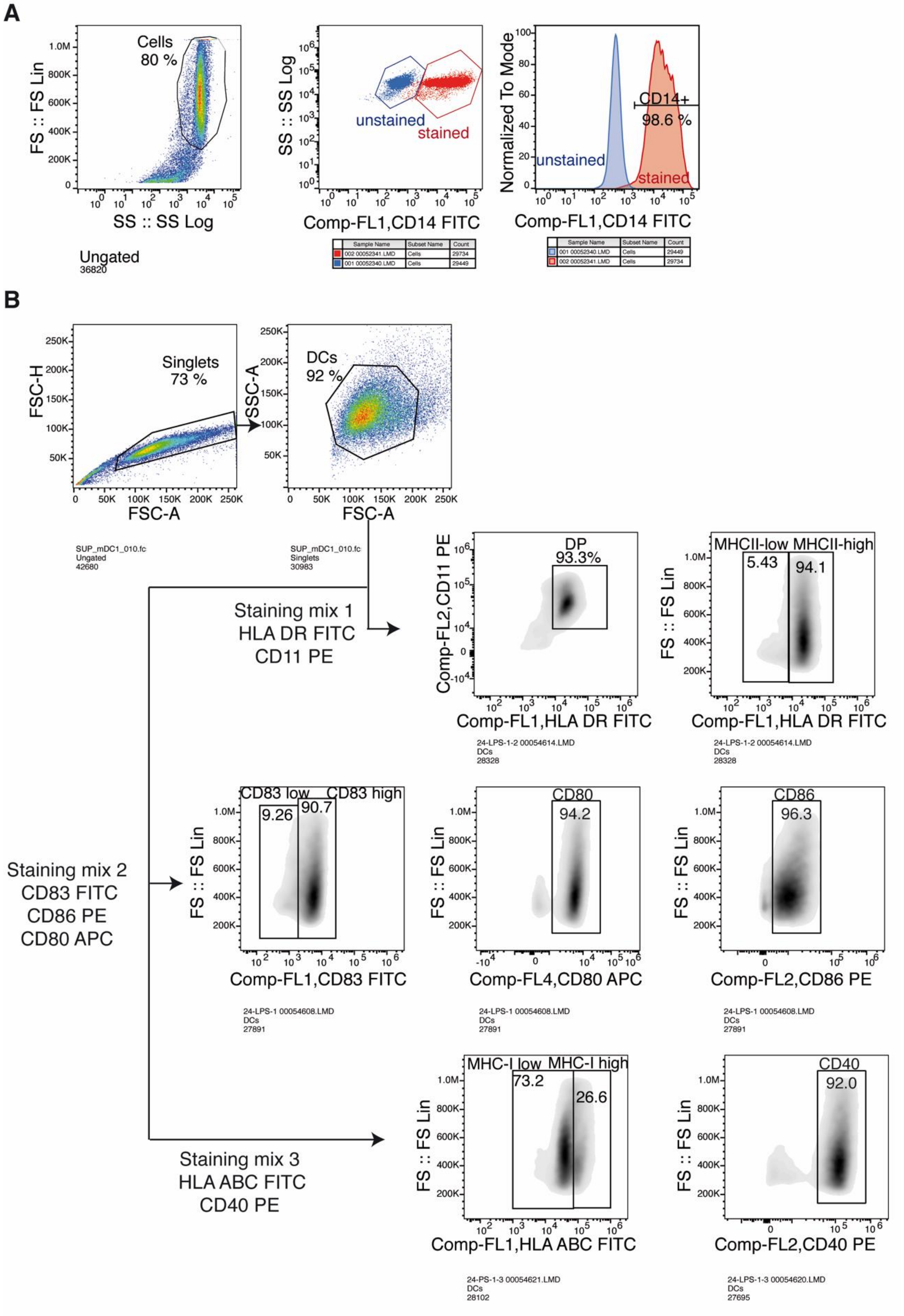

Figure S1. Monocyte CD14 staining and DCs gating and staining strategy. (**A**) Monocytes were purified by positive selection with CD14-labeled beads. Eluted cells were stained with an anti-human CD14-FITC antibody and analyzed by flow cytometry. Cells were gated from SS vs SS histogram and stained (red) and unstained (blue) cells profiles were compared. Forward vs Scatter histogram (left), Scatter vs CD14 expression profile (middle) and histogram (right) comparative. (**B**) After differentiation and different treatments DCs were subjected to three different staining mixes that contain different antibodies. Cell populations were selected by singlets selection and Scatter vs Forward gating. DCs were gated. Representative histograms are showed.

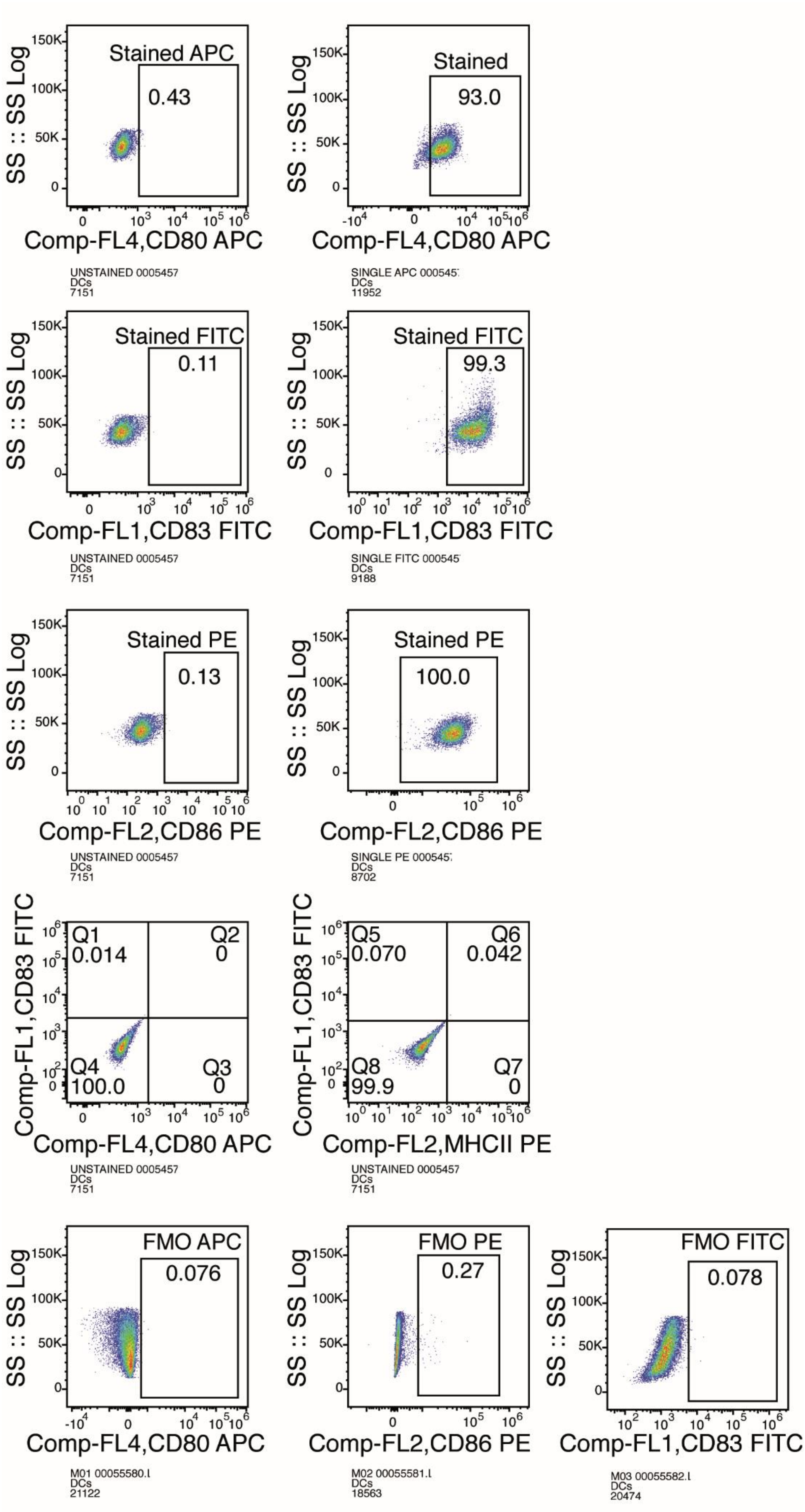
Stained APC
0.43
SS :: SS Log
Comp-FL4,CD80 APC
UNSTAINED 0005457
DCs
7151
Stained
93.0
SS :: SS Log
Comp-FL4,CD80 APC
SINGLE APC 000545
DCs
11952
Stained FITC
0.11
SS :: SS Log
Comp-FL1,CD83 FITC
UNSTAINED 0005457
DCs
7151
Stained FITC
99.3
SS :: SS Log
Comp-FL1,CD83 FITC
SINGLE FITC 000545
DCs
9188
Stained PE
0.13
SS :: SS Log
Comp-FL2,CD86 PE
UNSTAINED 0005457
DCs
7151
Stained PE
100.0
SS :: SS Log
Comp-FL2,CD86 PE
SINGLE PE 000545
DCs
8702
Q1
0.014
Q2
0
Q4
100.0
Q3
0
Comp-FL1,CD83 FITC
Comp-FL4,CD80 APC
UNSTAINED 0005457
DCs
7151
Q5
0.070
Q6
0.042
Q8
99.9
Q7
0
Comp-FL1,CD83 FITC
Comp-FL2,MHCII PE
UNSTAINED 0005457
DCs
7151
FMO APC
0.076
SS :: SS Log
Comp-FL4,CD80 APC
M01 00055580.
DCs
21122
FMO PE
0.27
SS :: SS Log
Comp-FL2,CD86 PE
M02 00055581.
DCs
18563
FMO FITC
0.078
SS :: SS Log
Comp-FL1,CD83 FITC
M03 00055582.
DCs
20474

Figure S2. Cell staining controls. To determine marker positivity, comparison of unstained and single stained cell profiles was performed. For triple staining the FMO was used. Representative histograms are shown.

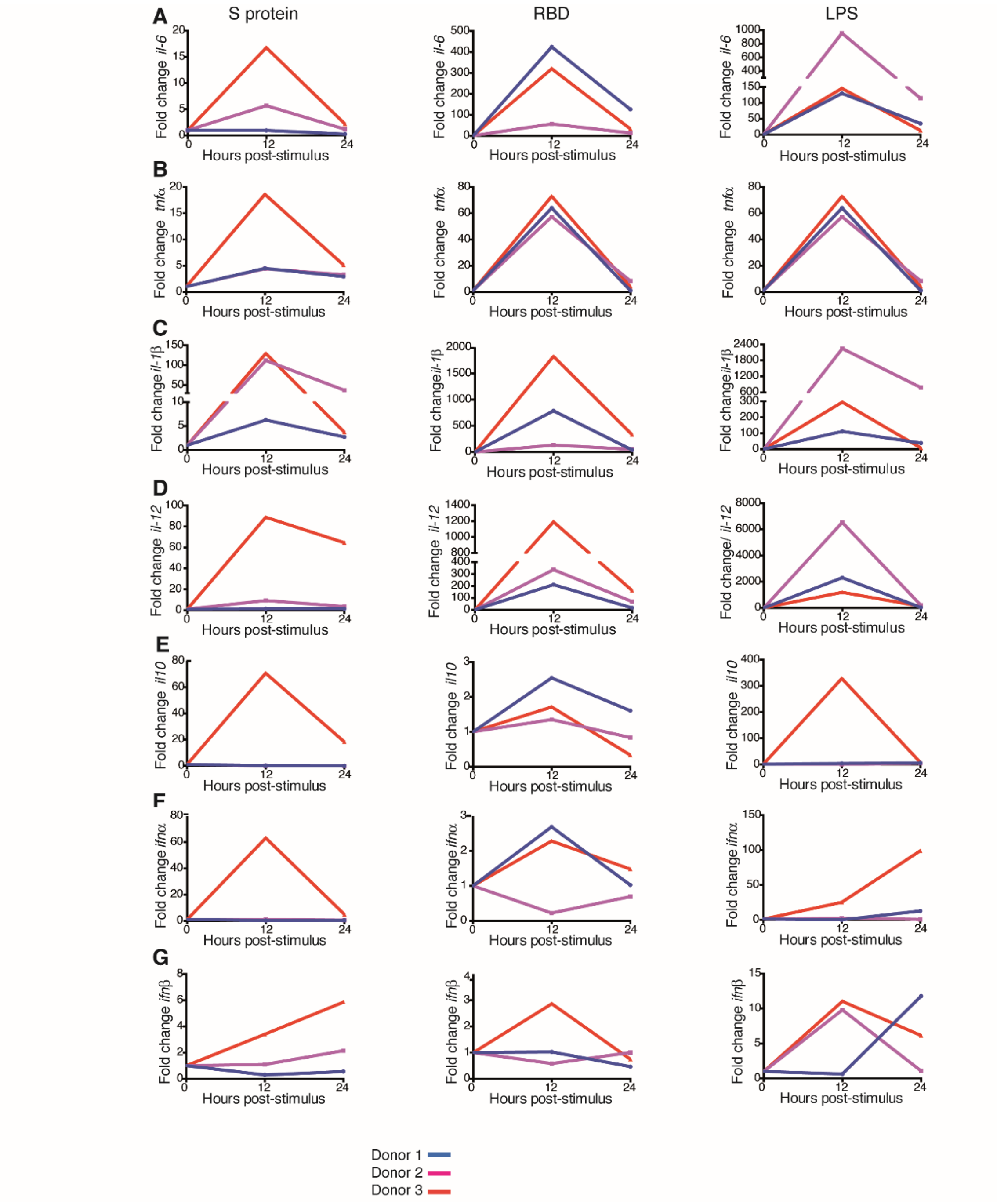

Figure S3. Spike protein and the RBD of SARS-CoV-2 induce a proinflammatory activation program in iDCs. Cells were incubated with indicated stimulus for 12 or 24 h, relative gene expression of different cytokines was evaluated by real-time PCR. Relative expression of IL-6 (**A**), TNFα (**B**), IL-1β (**C**), IL-12 (**D**), IL-10 (**E**), IFNα (**F**) and IFNβ (**G**) is shown. n = 3 different donors.

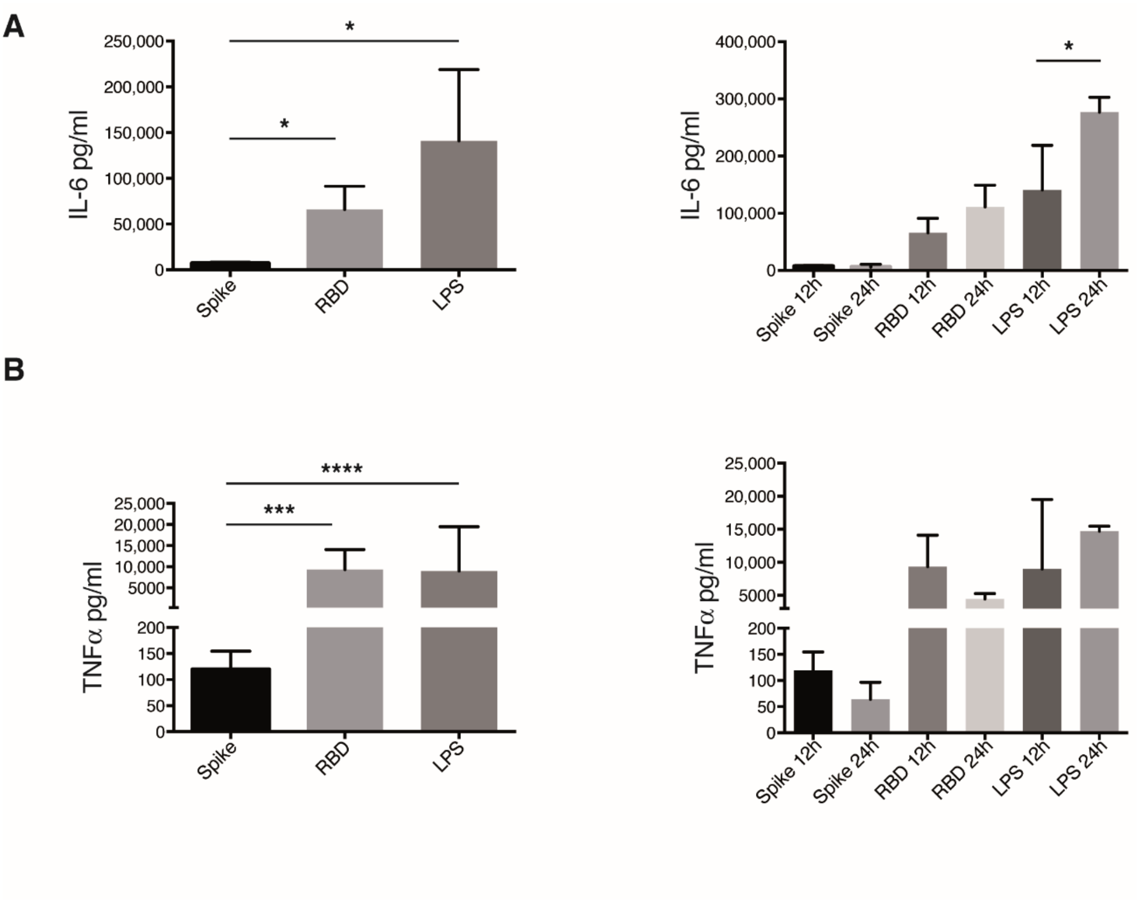

Figure S4. DCs IL-6 and TNFα production after 12 h and 24 h of Spike and the RBD of SARS-CoV-2 treatment. Cells were incubated with indicated stimulus and the concentration of the indicated cytokines secreted were measured in supernatants by ELISA. 12 h (left) and 12 h and 24 h (right) secretion graphics are shown. Multiple comparisons of the values of different treatments by using One-way ANOVA test. $n = 4$ donors in each case. The graphs show mean ± SEM; only significand differences are indicated. *$p < 0.05$; ***$p < 0.01$; ****$p < 0.006$.

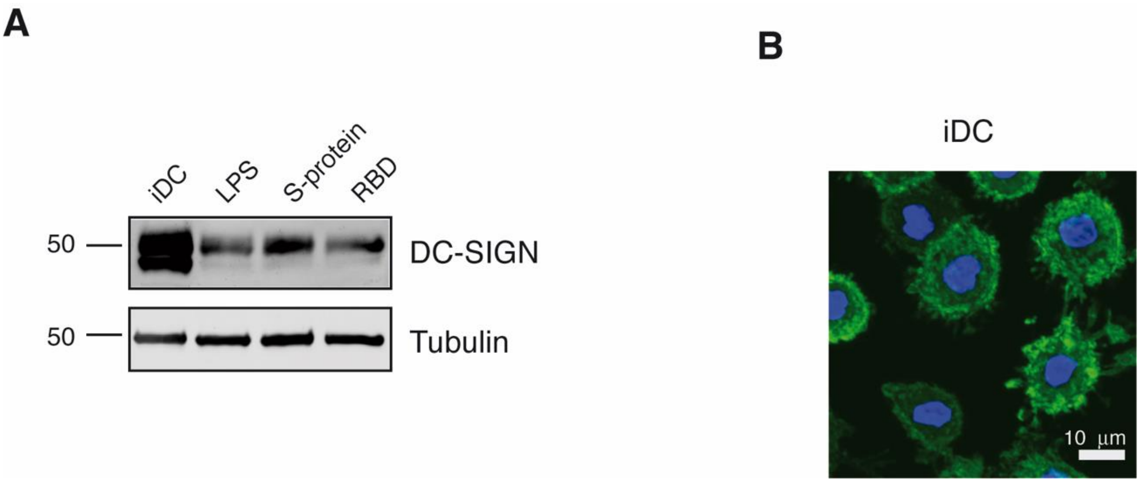

Figure S5. Dendritic cells express DC-SIGN. (**A**) Cells were incubated with indicated stimulus for the times indicated. Cells lysates were analyzed by immunoblot using in the indicated antibodies. (**B**) iDCs were fixed and analyzed by confocal microscopy to determine DC-SIGN expression. DC-SIGN (green) and nucleus (blue) in confocal images of one representative experiment is shown.

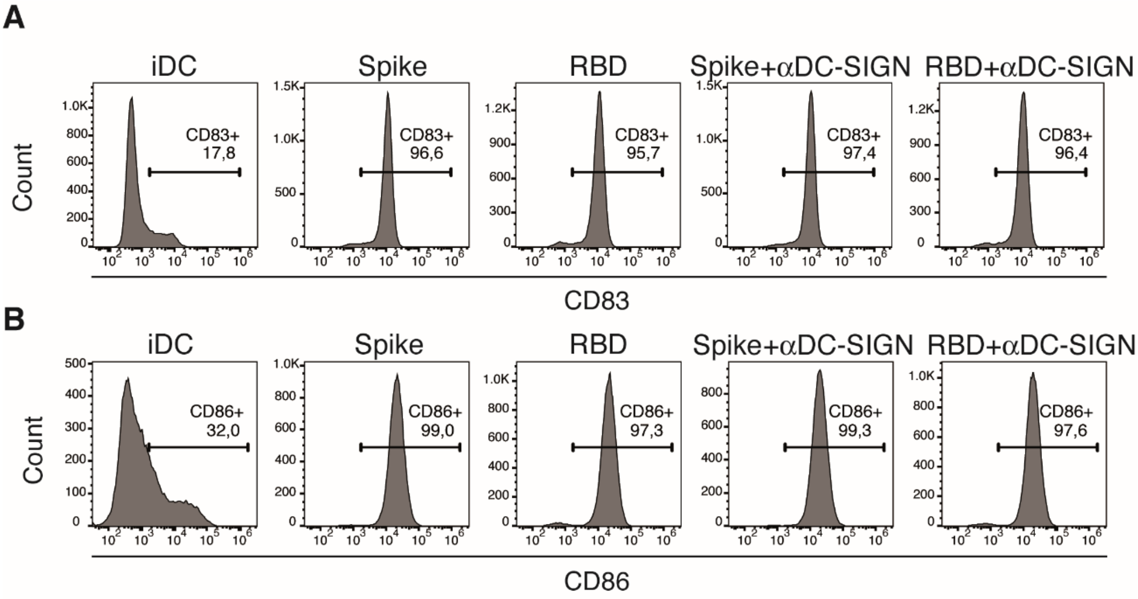

Figure S6. CD83 and CD86 expression histograms in DCs pre-treated with anti-DC-SIGN antibody followed by Spike and the RBD proteins stimulation. Cells were pre-incubated with a specific blocking anti-DC-SIGN antibody and stimulated for 24 h with the viral proteins. (**A**) CD83 and (**B**) CD86 expression profiles are shown. Histograms correspond to a representative experiment.
